# Success rates and prognosis of heart valvuloplasty and valve replacement performed for elderly patients

**DOI:** 10.12669/pjms.315.7583

**Published:** 2015

**Authors:** Weichao Liu, Fei He, Gongning Shi

**Affiliations:** 1 Weichao Liu, Department of Cardiothoracic and Vascular Surgery, Huaihe Hospital, Henan University, Kaifeng 475000, Henan Province, P. R. China; 2 Fei He, Department of Cardiothoracic and Vascular Surgery, Huaihe Hospital, Henan University, Kaifeng 475000, Henan Province, P. R. China; 3 Gongning Shi, Department of Cardiothoracic and Vascular Surgery, Huaihe Hospital, Henan University, Kaifeng 475000, Henan Province, P. R. China

**Keywords:** Heart valvuloplasty, Heart valve replacement, Elderly, Success rate, Prognosis

## Abstract

**Objective:**

To analyze the success rates and prognosis of heart valvuloplasty and valve replacement for elderly patients, and to provide clinical evidence.

**Methods:**

A total of 1240 patients who received heart valve surgeries in our hospital from June 2004 to October 2014 were selected and retrospectively analyzed. They were divided into two groups based on age (60), and those older than 60 (Group B) suffered from rheumatic valvular heart disease and nonrheumatic valvular heart disease including degenerative valve disease. Mitral valve replacement (MVR), tricuspid valve replacement (TVR), aortic valve replacement (AVR), double valve replacement (DVR), mitral valvuloplasty (MVP) and tricuspid valvuloplasty (TVP) were performed by using bioprosthetic and mechanical valves. Before surgery, coronary angiography, coronary artery bypass grafting (CABG), left atrial thrombectomy, left atrial wall folding and radiofrequency ablation were conducted. For the patients younger than 60 (Group A) who had congenital heart disease, rheumatic valvular heart disease and valvular heart disease, MVR, AVR, DVR, MVP, TVP and closed cuspid commissurotomy were performed with bioprosthetic and mechanical valves. The two groups were then monitored.

**Results:**

The mortality rates of Group A and Group B were 2.7% (16 cases) and 3.1% (20 cases) respectively. They died mainly of malignant ventricular arrhythmias, multiple organ failure, left ventricular rupture, low cardiac output syndrome, acute renal failure, respiratory failure, upper gastrointestinal bleeding, mechanical valve failure and cerebrovascular accident. The two groups had significantly different application rates of bioprosthetic valve, times of auxiliary ventilation and hospitalization stay lengths (P<0.05), but left ventricular ejection fractions, left ventricular end-diastolic diameters (LVEDDs), mortality rates as well as times of aortic cross-clamping and cardiopulmonary bypass were similar (P>0.05). LVEDD, complicated coronary artery disease, CABG and grade of the New York Heart Association Functional Classification were independent risk factors for postoperative death.

**Conclusion:**

When heart valvuloplasty and valve replacement were performed for elderly patients, the success rate and prognosis could only be improved by optimizing preoperative preparation, shortening the times of cardiopulmonary bypass and aortic cross-clamping, and paying particular attention to myocardial protection and postoperative treatment.

## INTRODUCTION

Recently, the elderly are more prone to hypertension, diabetes, obesity, renal failure and myocardial infarction due to lifestyle upgrade.^1^ The resulting weakened buffering capacity of vital organs, defensive ability and adaptability to surgery, as well as postoperative dysfunction of organs^2^ easily induce nervous system diseases such as arterial embolism and cerebral hypoperfusion. As one of the common clinical complications,^3^ valvular heart disease may even lead to death. Given organ dysfunction of elderly patients, the incidence of postoperative complications and mortality rate are often high.^4^ In this study, we analyzed the success rates and prognosis of heart valvuloplasty and valve replacement for elderly patients.

## METHODS

This study was approved by the ethics committee of our hospital, and written consent has been obtained from all patients. A total of 1240 patients who received heart valve surgeries in our hospital from June 2004 to October 2014 were selected and retrospectively analyzed. There were 644 males and 596 females, aged 32-76 years old (average: 54.3±10.6). They were divided into two groups according to the age of 60, and those older than 60 (Group B) suffered from rheumatic valvular heart disease and nonrheumatic valvular heart disease including degenerative valve disease. The patients younger than 60 (Group A) had congenital heart disease, rheumatic valvular heart disease and valvular heart disease. The two groups had similar heart-related factors such as left ventricular ejection fraction (LVEF) and left ventricular end-diastolic diameter (LVEDD), complications such as hypertension, diabetes and atrial fibrillation, and baseline clinical data such as gender (P>0.05).

### Inclusion criteria

Before surgery, all patients received color Doppler ultrasonography to diagnose valvular heart disease, with clear indications for surgery.^5^ The diagnosis was confirmed after surgery.

### Diagnostic criteria

Coronary artery disease was diagnosed by coronary angiography or electrocardiogram together with symptoms, medical history and risk factors. Diabetes was defined as the intake of a diabetic diet or oral hypoglycemic agents or a FPG above 7 mM/L according to the new ADA criteria.^6^ Hypertension was defined as the intake of antihypertensive drugs or two measurements of the systolic/diastolic blood pressure above 160/95 mm Hg according to WHO/ISHguideline.^7^ Diabetes was diagnosed according to the criteria issued by the Chinese Diabetes Society^8^ respectively. The New York Heart Association Functional Classification was used for grading.^9^ Arrhythmia or renal dysfunction was diagnosed by 12-lead electrocardiogram or serum creatinine and glomerular filtration rate.^10^

Under general anesthesia as well as medium, low-temperature cardiopulmonary bypass,^11^ surgery was performed through median incision, and then cold blood cardioplegia solution was perfused through the aortic root. For the patients whose aortic valves were not fully closed, direct perfusion after incising left and right coronary arteries or retrograde perfusion through the coronary sinus was employed, during which half of the cardioplegia solution was first given and the other half was given 0.5 h later. Ice crisps were, when necessary, put in the pericardial cavity for local cooling, and the right superior pulmonary veins were used to drain blood to the left atrium.^12^ Afterwards, the mitral valve in the left atrium was examined via the right atrium and interatrial septum. If there were lesions, the valve was cut off, but the subvalvular structure was retained. The valve was then continuously or intermittently mattress-sutured with 2-0 Prolene threads. As to the aortic valve, the blood vessel at aortic root was cut half-open, and the valve with lesions was disconnected before intermittent mattress suture.^13^ For tricuspid valve replacement (TVR), intermittent mattress suture was also employed, and fixing with artificial annular ring and intermittent mattress suture were used for mitral valvuloplasty (MVP). Tricuspid valvuloplasty (TVP) was carried out with DeVega technique. In the meantime, coronary artery bypass grafting (CABG) was performed to allow distal anastomosis, and proximal anastomosis was then realized after valve replacement.^14^ In this study, there were 612 cases of mitral valve replacement (MVR), 2 cases of TVR, 244 cases of aortic valve replacement (AVR), 241 cases of double valve replacement (DVR), 48 cases of MVP and 149 cases of TVP. Meanwhile, 122 cases of CABG, 96 cases of left atrial wall folding and 205 cases of left atrial thrombectomy were conducted.

### Statistical analysis

All data were analyzed by SPSS 17.0. The categorical data were expressed as (x±s) and inter-group comparisons were performed with t test. The numerical data were subjected to Chi-square test. Independent risk factors were found by using the Logistic regression method. P<0.05 was considered statistically significant.

## RESULTS

### Postoperative clinical data

**Fig.1:**
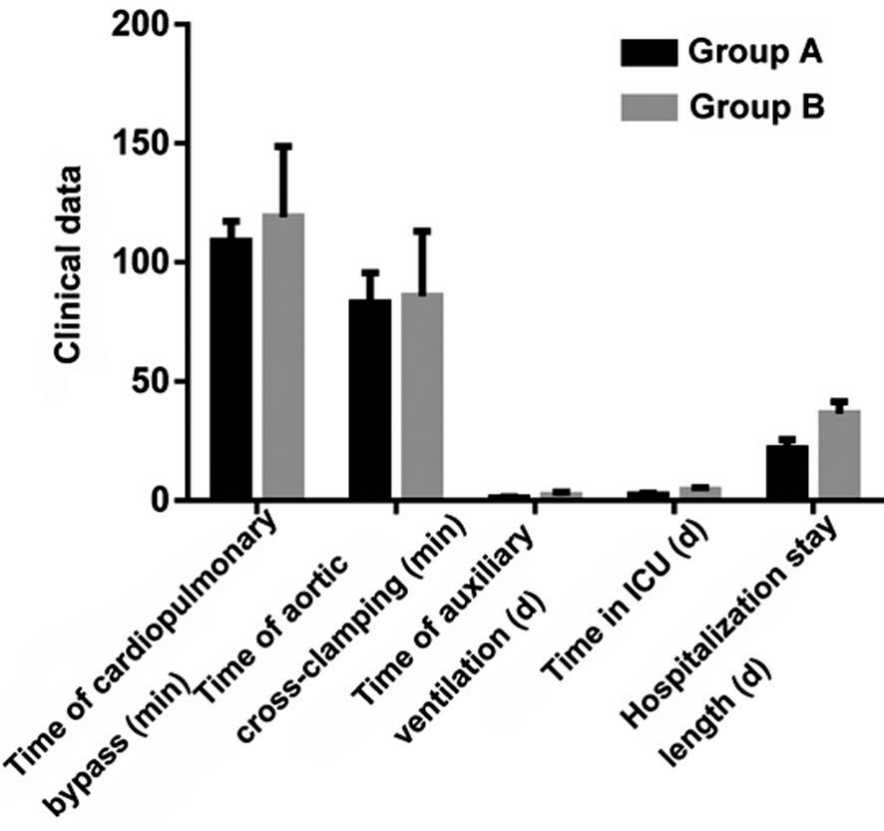
Postoperative clinical data.

The two groups had significantly different application rates of bioprosthetic valve, times of auxiliary ventilation and hospitalization stay lengths (P<0.05), but their mortality rates as well as times of aortic cross-clamping and cardiopulmonary bypass were similar (P>0.05) (Table-I and Fig.1).

**Table-I:** Postoperative clinical data.

Clinical data	Group A (603 cases)		Group B (637 cases)	
	Case number	Percentage	Case number	Percentage
Application rate of bioprosthetic valve	17	2.8%	80	12.6%
Morality	16	2.7%	20	3.1%

### Complications and mortality rates

The mortality rates of Group A and Group B were 2.7% (16 cases) and 3.1% (20 cases) respectively. They died mainly of malignant ventricular arrhythmias, multiple organ failure, left ventricular rupture, low cardiac output syndrome, acute renal failure, respiratory failure, upper gastrointestinal bleeding, mechanical valve failure and cerebrovascular accident. The incidence of postoperative complications such as cerebrovascular accident, respiratory failure and malignant ventricular arrhythmias as well as reoperation were significantly different (P<0.05) (Table-II).

**Table-II:** Complications and mortality rates (n, %).

Clinical data	Group A (603 cases)		Group B (637 cases)		P value
	Case No.	Morality rate	Case No.	Morality rate	
Respiratory failure	12	8.3%	37	5.4%	0.000
Acute renal failure	6	16.7%	9	22.2%	0.427
Cerebrovascular accident	5	0.0%	13	7.7%	0.042
Reoperation	15	0.0%	30	0.0%	0.030
Multiple organ failure	6	100.0%	9	55.6%	0.427
Low cardiac output syndrome	7	28.6%	16	12.5%	0.053
Mechanical valve failure	8	12.5%	4	0.0%	0.127
Malignant ventricular arrhythmias	7	42.9%	17	17.6%	0.035
Upper gastrointestinal bleeding	10	10.0%	14	7.1%	0.456
Poor wound healing	16	0.0%	18	0.0%	0.861
Left ventricular rupture	3	33.3%	3	33.3%	0.959

### Multivariate regression analysis of death-related risk factors

LVEDD, complicated coronary artery disease, CABG and grade of the New York Heart Association Functional Classification were independent risk factors for postoperative death, as suggested by multivariate regression analysis (Table-III).

**Table-III:** Multivariate regression analysis of death-related risk factors.

Variable	Chi-square value	P value	OR value	OR 95% confidence interval
LVEDD	5.208	0.020	5.403	0.12871~3.06308
Coronary artery disease	4.648	0.028	3.274	0.10107~2.21471
CABG	6.114	0.0121	0.712	0.61523~5.33455
Grade of the New York Heart Association Functional Classification	6.602	0.011	0.466	-0.95865~-0.12013

## DISCUSSION

Elderly patients are vulnerable to brain, lung, liver and renal dysfunctions owing to long disease course,^15^ and long-term cardiac injury easily induces arrhythmia and damages livers and lungs. If the patients who are planning to receive surgery for valvular heart disease are complicated with hypertension, diabetes and coronary artery disease, the incidence rates of postoperative complications and morality rates are bound to increase. Therefore, it is of great significance to perform coronary angiography for these patients to clarify surgical indications and to evaluate the functions and states of vital organs. Moreover, diuretic, cardiac drugs together with sufficient nutritional support are necessary to lighten the load on the heart and to augment the tolerance to surgery.^16^

In this study, surgery was simplified and success rate was elevated by using general anesthesia and low-temperature cardiopulmonary bypass and by completely exposing the heart and blood vessels. Since elderly patients are most prone to left atrial thrombi,^17^ it is also important to eliminate them and to timely remove the envelope around them. Mild hypothermia while the heart is maintained beating or interventional therapy is highly recommended for the high-risk elderly with poor cardiopulmonary function and those complicated with other diseases, so as to decrease the postoperative mortality rate, to improve the cardiac function, to relieve ventricular remodeling, and to increase the success rate, with safety, minor traumas and short hospitalization stay also.^18^

For the sake of the elderly with valvular heart disease, the surgical method should be cautiously selected, the time of aortic cross-clamping should be minimized, the myocardium should be well protected, and particular attention should be paid to postoperative treatment.^19^ For patients with poor cardiopulmonary function and considerable complications, CABG was performed simultaneously and auxiliary ventilation was used. Nevertheless, the treatment time and hospital stay were extended, accompanied by increased incidence of postoperative complications and mortality rates. Furthermore, experienced surgeons are required for these patients, given the long disease course, low adaptability of the myocardium and low tolerance to hypoxia and ischemia.

### Limitations of the study

In such a study design, selection of the population may introduce important biases in the findings. Two-thirds of our patients had ischemic symptoms (whether chronic or unstable angina) requiring coronary evaluation and do not represent a standard diabetic population. Coronary angiography has its own limitations in the study of coronary atherosclerosis. It may underestimate the early development of atheroma since compensatory enlargement of the vessel wall may initially accommodate young atheromatous plaques with limited lumen deformation.

In summary, heart valvuloplasty and valve replacement are safe and reliable for elderly patients with valvular heart disease, ensuring satisfactory prognosis.
